# Characterization and Release Behavior of a Thiosemicarbazone from Electrospun Polyvinyl Alcohol Core‒Shell Nanofibers

**DOI:** 10.3390/polym12071488

**Published:** 2020-07-03

**Authors:** Hossein Barani, Mohsen Khorashadizadeh, Alexander Haseloer, Axel Klein

**Affiliations:** 1 Department of Carpet, Faculty of Arts, University of Birjand, Birjand 9717434765, Iran; 2 Department of Medical Biotechnology, Birjand University of Medical Sciences, Birjand 9717853577, Iran; khorashadi@bums.ac.ir; 3 Department of Chemistry, Institute for Inorganic Chemistry, University of Cologne, Greinstrasse 6, D-50939 Cologne, Germany; ahaseloe@smail.uni-koeln.de

**Keywords:** PVA, fiber mat, drug release profile, co-axial electrospinning, minimal inhibitory concentration

## Abstract

Mats of polyvinyl alcohol (PVA) core–shell nanofibers were produced using coaxial electrospinning in the presence of a thiosemicarbazone (TSC) *N*4-(S)-1-phenylethyl)-2-(pyridin-2-yl-ethylidene)hydrazine-1-carbothioamide (HapyTSCmB). Monolithic fibers with 0% or 5% TSC and core–shell fibers with 10% TSC in the spinning solution were studied to compare stability and release rates. SEM showed the formation of uniform, bead-free, cylindrical, and smooth fibers. NMR spectroscopy and thermal analysis (TG/DTA) gave proof for the chemical integrity of the TSC in the fiber mats after the electrospinning process. Attenuated total reflection Fourier-transform infrared (ATR-FTIR) spectroscopy showed no TSC on the surface of the PVA/TSC-PVA fibers confirming the core–shell character. The TSC release profiles of the fibers as studied using UV-vis absorption spectroscopy showed a slower release from the PVA/TSC-PVA core–shell structure compared with the monolithic PVA/TSC fibers as well as lower cumulative release percentage (17%). Out of several release models, the Korsmeyer–Peppas model gave the best fit to the experimental data. The main release phase can be described with a Fick-type diffusion mechanism. Antibacterial properties were tested against the Gram-positive *Staphylococcus aureus* bacterium and gave a minimal inhibitory concentration of 12.5 μg/mL. 3-(4,5-dimethyl-2-thiazolyl)-2,5-diphenyl-2*H*-tetrazoliumbromide (MTT)-based cytotoxicity experiments showed that the cell viability of fibroblast at different contents of TSC was slightly decreased from 1.5% up to 3.5% when compared to control cells.

## 1. Introduction

**Scheme 1:**
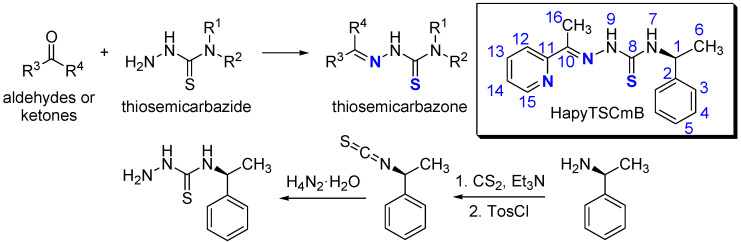
General synthesis of thiosemicarbazones (TSC) from thiosemicarbazides. Top right: the title TSC: *N*4-(*S*)-1-phenylethyl)-2-(pyridin-2-yl-ethylidene)hydrazine-1-carbothioamide (HapyTSCmB) with numbering.

Thiosemicarbazones (TSCs) and their metal complexes are versatile molecules with a wide spectrum of antimicrobial, antitrypanosomal, antitumor, antimalarial, and antiviral activities and have been studied intensely in pharmaceutical and medicinal chemistry [1,2,3,4,5,6,7,8,9,10,11,12,13]. TSCs are able to block the genetic expression of wide variety of viruses [11,13], including the herpes simplex virus [14,15,16,17,18,19,20,21]. This virus affects the external genitalia, anal region, mucosal surfaces, and skin in other parts of the body, and external treatment is possible. This brought us to the idea to incorporate a TSC into fibrous mats to be used for treating herpes simplex infections. Very beneficial for the use of TSC in medicine is the comparably simple and modular synthesis from thiosemicarbazides and aldehydes or ketones (Scheme 1) allowing a plethora of structural variations.

2-Acetyl-pyridine thiosemicarbazones have been studied since the 1960s for their interesting biological activities, and they stood also at the cradle when TSC were first investigated against herpes simplex viral infections [18,19,20,21]. Therefore, we decided to study the new TSC *N*4-(S)-1-phenylethyl)-2-(pyridin-2-yl-ethylidene)hydrazine-1-carbothioamide (HapyTSCmB), which complies with the 2-acetyl-pyridine structural motif and contains the chiral methyl-benzyl amine end group.

Drug solubility and bioavailability are the serious challenges in the field of pharmaceuticals formulation [22,23,24,25,26]. Especially for hydrophobic drugs, solubility improvement is the key issue in the development of formulations [23]. Several solubility improvement methods such as solid dispersion, complexation, lipid-based systems, micronization, nanonization, and co-crystallization have been reported for improving the solubility and bioavailability of poorly water-soluble drugs [27]. Among them, the solid dispersion is the most widely used method in formulation development [26]. The electrospinning method represents a combination of solid dispersion technology and nanotechnology. In this method, solid fibers in the micro- or nanometer range are produced from a polymeric fluid stream or melt delivered through a millimeter-scale nozzle [22,23,24,25,26,27,28,29,30]. In the pharmaceutical field, electrospinning has recently gained high interest for the production of drug delivery systems based on nanofibers [31,32,33,34,35]. There are different approaches for loading drugs in electrospun fibers to obtain a controlled drug delivery system. These include the following: (1) blending of the drug in the electrospinning polymer solution; (2) adsorption of drug molecules on the surface of electrospun fibers through physical interactions; (3) emulsion electrospinning, and (4) co-axial electrospinning [32,36,37,38]. The blending of drugs with the appropriate polymeric solution remains the most predominant method and is simple compared to others, but some requirements should be met in order to gain the desired results [36,37,38]. e.g., in the case of insufficient solubility of the drug in the polymer solution, phase separation can lead to the agglomeration of drug molecules on the surface of the fiber and lead to unwanted burst release [39,40,41,42,43,44]. Therefore, a good match of hydrophilic and hydrophobic properties between drugs and polymers is very important for blending electrospinning [36,37,38,39,40,41,42,43,44,45]. e.g., polyvinyl pyrrolidone (PVP) nanofibers have been reported to improve the solubility of some hydrophobic drugs [46]. Polyvinyl alcohol (PVA) is a synthetic semi-crystalline hydrophilic polymer [47,48] that has already been explored for drug delivery applications due to its solubility in water. PVA is biodegradable and biocompatible, and water-soluble electrospun fibers can be formed from an aqueous solution [49,50,51,52].

The drug release behavior is highly dependent on the distribution of the drug molecules in the nanofibers as well as on the morphology of the nanofibers. For achieving perfect encapsulation, polymers should ideally be chosen for perfect compatibility/miscibility with the hydrophilic or lipophilic drugs. However, recent studies have shown that coaxial electrospinning allows also the manufacturing of core–shell nanofibers with homogeneously dispersed drugs of drug/polymer combinations with low compatibility/miscibility [45,46,53,54,55,56,57]. Another big advantage of this technique is that the shell provides protection to the core-loaded drugs through shielding it against the external environment. Thus, slow removal of the shell allows the sustained release of drugs even if they are quite sensitive or unstable in a biological surrounding.

In this study, we used a coaxial electrospinning setup for producing core–shell PVA/TSC-PVA and monolithic PVA/TSC fiber mats for releasing the poorly water soluble and hydrolysis-sensitive pyridine based TSC *N*4-(S)-1-phenylethyl)-2-(pyridin-2-yl-ethylidene)hydrazine-1-carbothioamide (HapyTSCmB, Scheme 1). Insufficient solubility of the quite hydrophobic drug in the hydrophilic PVA polymer solution might result in a phase separation leading to the agglomeration of drug molecules on the surface of the fiber with a direct contact of the biomolecule with the external environment. Therefore, the monolithic PVA/TSC fibers might either contain the TSC molecules homogeneously distributed in the PVA polymer, or the TSC might be segregated close to the surface of the fibers. In contrast to this, the core–shell approach for the preparation of PVA/TSC-PVA fibers proposes to shield the drug-containing core against rapid chemical or biological decomposition/degradation and also stabilizes mechanically the fragile core. The effect of the TSC content on the morphology of these two different fibers and the release properties were investigated in detail.

## 2. Materials and Methods

### 2.1. Materials

Polyvinyl alcohol (PVA) (*M*_w_ 146,000–186,000, 99% hydrolyzed, Merck Co., Darmstadt, Germany), dimethyl sulfoxide (DMSO-d_6_, Sigma-Aldrich, Taufkirchen, Germany), and phosphate-buffered saline (PBS) (Fisher Scientific, city, Germany) were purchased and used without further purification. First, 4 g of *N*4-(*S*)-1-phenylethyl)-2-(pyridin-2-yl-ethylidene)hydrazine-1-carbothioamide (HapyTSCmB) was synthesized from 1-(pyridin-2-yl)ethan-1-one and (*S*)-*N*4-(1-phenylethyl)hydrazinecarbothioamide (thiosemicarbazide) in a process derived from an established ultra-sonic assisted condensation method by Carradori et al. [11] (Scheme 1) in 74% yield. Elemental analysis calculated for C_16_H_18_N_4_S (298.41 g/mol): C 64.40, H 6.08, N 18.77, S 10.75%; found: C 64.27, H 6.05, N 18.68 S 10.70%. ^1^H NMR (DMSO-d_6_, 300 MHz) *δ* [ppm] = 10.41 (s, 1H, NH9), 8.70 (d, 1H, *J* = 8.8 Hz, NH7), 8.59 (d, 1H, *J* = 4.7 Hz, H15), 7.84 (t, 1H, *J* = 8.6 Hz, H13), 7.32–7.44 (m, 5H, H3, H4, H5), 7.24 (t, 1H, *J* = 7.2 Hz, H14), 5.76 (quint, 1H, *J* = 7.0 Hz, H1), 2.41 (s, 3H, H16), 1.58 (d, 3H, *J* = 7.0 Hz, H6), (for numbering see Scheme 1). EI-MS(+) m/z = 299 [M+H]^+^.

### 2.2. Characterization of the Polymer Solutions

The rheological performance of the PVA and PVA/TSC solutions (PVA, PVA/TSC5%, and PVA/TSC10%) in water–ethanol (9:1) was studied using a rheometer AR-G2 (TA instruments, Newcastle, DE, USA) with a parallel plate setup (20 mm diameter) at 25 °C and shear rates up to 500 s^−1^. A Mettler Toledo FiveGo conductivity meter (Mettler Toledo, Gießen, Germany) was used to study the electrical conductivity of the above polymer solutions. The conductivity of each polymer solution sample was measured for at least 10 runs and reported as average conductivity (µS/cm).

### 2.3. Fabrication of TSC-Loaded Monolithic and Core–Shell PVA Fibers

**Scheme 2:**
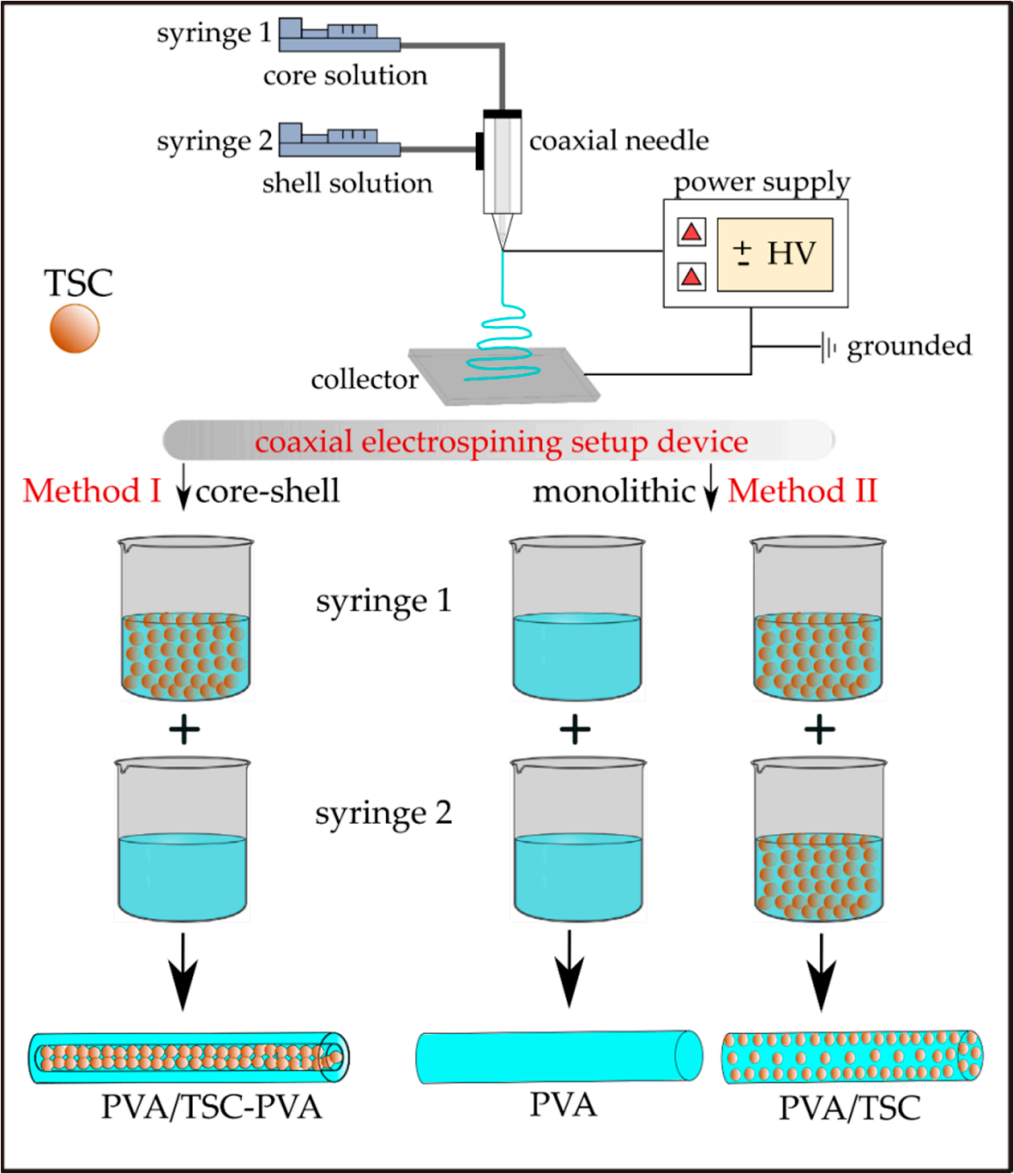
Preparation of HapyTSCmB loaded core–shell (PVA/TSC-PVA) and monolithic (mono-PVA/TSC) fibers with a coaxial electrospinning device. PVA: polyvinyl alcohol.

The loading of HapyTSCmB into the PVA fibers was carried out using two different electrospinning procedures (Scheme 2). Using a coaxial electrospinning setup device allowed adding the TSC to the PVA core of the fibers, which is surrounded by a PVA shell (Method I; PVA/TSC10-PVA). The same setup was used to produce monolithic PVA (Method II; PVA/TSC5). The process parameters are summarized in Table 1.

Prior to the fabrication of the fibers, we performed a series of preliminary tests varying polymer concentration, flow rate, and applied voltage to optimize the parameters for ensuring a stable electrospinning jet. According to this, polymer solutions (8 wt% based on the final solid content) for core and shell were prepared by dissolving (1.6 g) PVA polymer in 18.89 mL water–ethanol solution (9:1) under stirring (750 rpm) on a hot plate for at least 24 h at 50–55 °C. HapyTSCmB (0, 5, and 10 wt% relative to PVA) was added to the polymer solutions. A needle with a gauge of 14 (OD 2.00 mm) was used for the shell spinneret, and a smaller needle (gauge 22, OD 0.70 mm) was used for the core spinneret for the core–shell fibers. The polymer solutions were delivered to the spinnerets by two syringe pumps (LA-100, Landgraf Laborsysteme, Langenhagen, Germany) at feeding rates of 0.3 mL/h. The fibers were collected on an Al foil placed at a distance of 15 cm from the needle tip. The electrospinning process was performed at an applied voltage of 13 kV supplied by a DC high-voltage power supply (FuG Elektronik GmbH, Schechen, Germany).

### 2.4. Characterization of the Fibers

The surface morphology of the electrospun monolithic PVA and PVA/TSC5 and the core–shell PVATSC10-PVA fibers was characterized using scanning electron microscope (SEM) (Zeiss Neon40 CrossBeam, Zeiss, Oberkochen, Germany). The samples were directly attached on the sample holder with no coating pre-treatment. The average diameter of each sample was determined using the Digimizer 4.1.1.0 software with at least 100 measurements performed for each electrospun fiber. The characterization of the core–shell structure of the fibers was attempted using transmission electron microscopy (TEM) (LEO 912 Omega, Zeiss, Oberkochen, Germany) operated at 120 kV with zero loss conditions. The samples for TEM were prepared by collecting the nanofibers on carbon-coated copper grids. The thermal behavior of the fibers was studied using thermogravimetry/differential thermal analysis (STA 1500, Bähr Thermoanalyse GmbH, Hüllhorst, Germany) in the range of 30 to 600 °C at a heating rate of 10 °C/min under N_2_ atmosphere, while the thermal stability of the TSC was studied using a TGA/DSC1 (Mettler Toledo, Gießen, Germany) instrument under the same conditions. Nuclear magnetic resonance (NMR) spectroscopy on pieces of the fiber mats dissolved in DMSO-d_6_ were recorded at room temperature on a Bruker Avance II AV300 (300 MHz), using a triple resonance ^1^H, ^n^BB inverse probe head (Bruker, Karlsruhe, Germany). Fourier-transform infrared (FTIR) spectra were recorded in the range of 4000 to 500 cm^−1^ using in a PerkinElmer Spectrum 400 instrument (PerkinElmer, Rodgau, Germany) in attenuated total reflection (ATR) mode. Samples were directly placed into the device with no pre-treatment and recorded with 4 scans and a resolution of 4 cm^−1^ at room temperature (20 °C). The absorption spectra were normalized based on CH_2_ bending vibration (1427 cm^−1^) [58].

### 2.5. In Vitro Release

A UV-vis spectrophotometer (Varian Cary, 50 Scan, Varian, Darmstadt, Germany) was used to study the TSC release from the monolithic PVA/TSC5 and from the core–shell PVA/TSC10-PVA fibers. First, 1 mg of each dried mat was soaked in a glass bottle with phosphate buffer solution (PBS = 10 mL, pH = 7.4). The bottle was tightly capped and immersed in a shaking incubator bath at 37 °C at 100 rpm. At each predetermined interval (10, 20, 30, 40, 60, 120, and 240 min), a 4 mL sample was collected, and an equal volume of buffer was added to retain the initial volume. The absorbance of HapyTSCmB in the medium was determined at a wavelength of 260 nm, and the released TSC amount was determined from a calibration curve of pure TSC in PBS at concentrations ranging from 0.023 to 0.75 µg mL^−1^. The averaged results of three measurements were recorded for each mat sample. The cumulative release percentage of TSC was determined as (*m*_t_/*m*_0_ × 100), in which *m_t_* is the mass of TSC released up to the time *t*, and *m_0_* is the initial amount of the loaded TSC. The release kinetics of HapyTSCmB from the PVA electrospun mats were studied by fitting the experimental release data to mathematical drug release models such as zero-order, first-order, Higuchi, and Korsmeyer–Peppas.

### 2.6. In Vitro Antibacterial Assay

The minimal inhibitory concentration (MIC) of the TSC was examined by preparing different concentrations (6.25, 12.5, 25.0, 50.0, and 100 μg/mL) by serial dilution of the prepared stock solution. First, 1 mg of TSC was dissolved in 100 μL DMSO for preparing the stock solution [59]. The in vitro antibacterial activity of the TSC against the Gram-positive bacterium *Staphylococcus aureus* (ATCC 6538) was studied using the agar well diffusion method [60]. The surface of preloaded Petri dishes containing Mueller–Hinton agar was seeded with bacterial suspensions (equivalent to 0.5 McFarland standard, 1.5 × 10^8^ CFU/mL), using a sterile cotton swab. After that, wells with 6 mm diameter were created by punching a stainless steel cylinder onto the agar plates and removing the agar to form a well. Finally, 70 mL aliquots of each prepared sample were placed individually in the well. The minimal inhibitory concentrations (MIC, μg/mL) were recorded as the lowest concentration of TSC in the medium that showed no microbial growth by visual observation. The DMSO-wetted disk was used as control and run simultaneously.

### 2.7. In Vitro Cytotoxicity

***Cell culture.*** Human fibroblast cells were obtained from the cell bank of the Stem Cell Research Center (Tehran, Iran). The cells were cultured in complete DMEM (Dulbecco’s Modified Eagle medium) supplemented with 10% FBS (Fetal Bovine Serum), 100 units/mL penicillin, and 100 mg/mL streptomycin and incubated at 37 °C in humidified atmosphere containing 5% CO_2_.

***MTT assay.*** Fibroblast cells were seeded in 96-well plates at a density of 3 × 10^3^ cells/well and incubated overnight for 24 h. After that, they were treated with varying amounts of HapyTSCmB (12.5–50 μg/mL). After 48 h, the cell viability was measured using the 3-(4,5-dimethyl-2-thiazolyl)-2,5-diphenyl-2*H*-tetrazoliumbromide (MTT) assay. Briefly, 20 µL of MTT (5 mg/mL) reagent was added to each well and incubated for 4 h. Then, the supernatant was removed, and the furmazan crystals were dissolved in 100 µL of DMSO. The absorbance was recoded at 570 nm using a multi-plate reader (Epoch, Biotek, Vinooski, VR, USA). The cell viability was calculated by dividing the absorbance of treated cells into the absorbance of the control cells.

## 3. Results and Discussion

### 3.1. Preparation and Characterization of the Fibers

**Figure 1:**
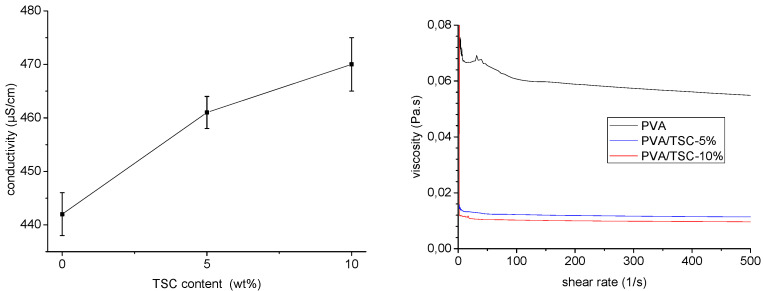
Conductivity (**left**) and viscosity (**right**) of PVA/TSC (0%, 5%, and 10%) polymer solutions in water–ethanol (9:1).

In initial experiments, the conductivity and viscosity of polymer solutions for electrospinning containing PVA in water–ethanol solution (9:1) and various amounts of the thiosemicarbazone HapyTSCmB (*N*4-(*S*)-1-phenylethyl)-2-(pyridin-2-yl-ethylidene)hydrazine-1-carbothioamide, Scheme 1) were measured (Figure 1). The conductivity and viscosity of electrospinning polymer solutions have a strong impact on the morphology and diameter of PVA fibers [36,37,61,62,63,64]. We found that adding 10 wt% of the TSC to the PVA solution caused a slight increase (approximately 6.5%) in conductivity and a significant decrease of about 83% in the viscosity (Figure 1). Compared to the pristine polymer solution (0.061 Pa s), the viscosity was 0.012 and 0.010 Pa s for 5 and 10 wt% respectively. The viscosity of the polymer solution ultimately determines the electrospinnability, and fiber diameters increase as the viscosity of a polymer solution increases. However, at concentrations beyond a certain limit, the viscosity of the solution becomes exceedingly high, disrupting the flow of the polymer solution through the capillary [37,61,65]. Viscosity-reducing additives for reducing the diameter of electrospun fibers have been reported [66,67,68]. It was found that the additive reduces the average chain length and thus reduces the viscosity of the polymer solution and thus the fiber diameter [66]. In our experiments, the TSC turned out to be a viscosity-reducing additive in the PVA polymer solution.

**Figure 2:**
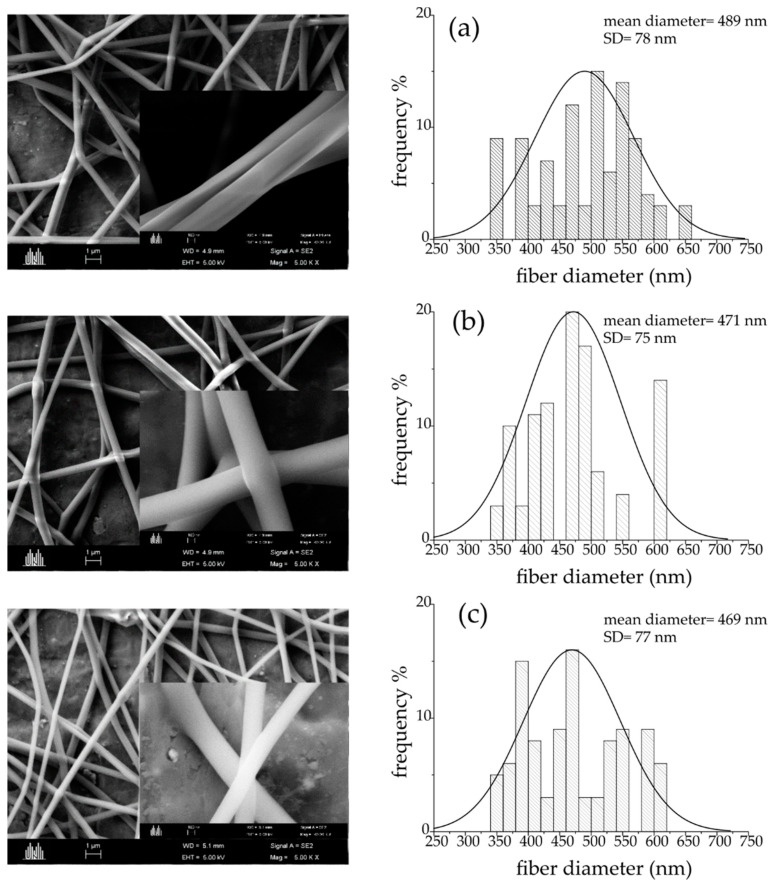
Scanning electron microscopy (SEM) of the monolithic PVA and PVA/TSC5, and the core–shell PVA/TSC10-PVA fibers (**left**) and their diameter distributions (**right**) with different TSC content (**a**) PVA, (**b**) PVA/TSC5, and (**c**) PVA/TSC10-PVA.

Scanning electron microscopy (SEM) of the monolithic PVA, PVA/TSC5, and the core–shell PVA/TSC10-PVA fibers show a uniform and bead-free cylindrical shape and smooth surfaces (Figure 2). The calculated average size of the pure PVA fibers showed an average diameter of 489 ± 78 nm. The addition of TSC resulted in a slight decrease of the average diameter (Figure 2). Adding 5 wt% of TSC in the core and shell resulted in average diameters of 471 ± 75 nm, while adding 10 wt% in the core only results in average diameters of 469 ± 77 nm, which represents a decrease of approximately 4% compared with pristine PVA. The reduction in size is in line with the reduced viscosity of the spinning solution through the added TSC.

Seeking to confirm the core–shell character of the PVA/TSC10-PVA fibers, we tried TEM (transmission electron microscopy) but failed to resolve the core from the shell. This is not unexpected, since the chemical compositions of the core and shell are very similar.

**Figure 3:**
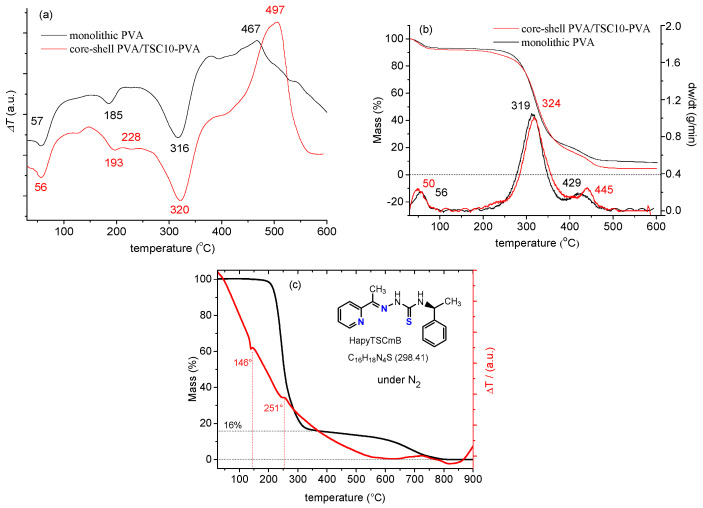
Thermograms of the monolithic PVA and the core–shell PVA/TSC10-PVA fiber mats (**a**) differential thermal analysis (DTA), (**b**) thermogravimetry/derivative thermogravimetry (TG/DTG); (**c**) TG/DTA of pure TSC.

We found characteristic endothermic and exothermic peaks (Figure 3) in the differential thermal analysis (DTA), thermogravimetry (TG), and derivative thermogravimetry (DTG) thermograms of the monolithic PVA and core–shell PVA/TSC10-PVA fibers. The data agree with literature data reporting three endothermic and one significant exothermic peak in the DTA curve of monolithic PVA [69,70]. For both fibers, the first endothermic peaks were observed at 56 or 57 °C in the DTA. They are probably due to the evaporation of physically adsorbed water, with a mass loss of about 10% (TG/DTG). At the same time, reported values for the glass transition (*T_g_*) of PVA range from 34 to 85 °C [70,71,72,73] and might overlap with the loss of water. Weaker endothermic peaks occurred at 185 °C for PVA, but at 193 °C for the core–shell fibers, both without mass loss. We assign them both to the melting transition (*T_m_*), the reported values for PVA are in the range from 180 to 240 °C depending on the degree of hydrolysis [51,70,71,72,73]. The higher *T*_m_ for the core–shell fibers seems to be due to some re-enforcement of the fibers either through the TSC or the core–shell structure. For the core–shell fibers, a plateau followed, peaking at 228 °C, which might be due to the partial melting and evaporation of the TSC. Indeed, the DTA of the TSC (Figure 3c) shows endothermic peaks at 146 °C and 251 °C for melting (no mass loss) and evaporation (mass loss of 84%), respectively. Thus, the intense endothermic peaks found for the fibers at 316 and 320 °C were attributed to evaporation of the TSC from the fibers, probably overlapping with the decomposition of the side chain of the PVA polymer. Finally, the exothermic peak at 467 (PVA) and 497 °C (PVA-TSC10-PVA) appears not only at different temperature, but is also far more pronounced for the core–shell fibers than for the monolithic PVA. Again, we conclude some stabilization effect of residual TSC. This is supported from the observation that the evaporation of TSC (mass loss) from the fibers continues after 500° for the fibers (Figure 3b).

The TG and DTG curves confirmed that the DTA peaks at 56 and 57 °C with a mass loss of about 10% are due to the elimination of the physically adsorbed water. Significant weight loss of about 60% occurred at 319 and 324 °C, confirming the supposed side chain decomposition of PVA after melting. At the lower offset of this peak, the core–shell fibers lose some weight at around 230 °C in line with the assumed partial evaporation of the TSC. This process overlaps partially with the final weight loss at 429 and 445 °C of PVA and core–shell PVA/TSC10-PVA fibers, respectively, which are due to the decomposition of the PVA main chain [72]. The overall mass losses accumulate to 91% for the monolithic PVA and 95% for the core–shell fibers. Summarizing, although the presence of the TSC modifies slightly the thermal properties on the core–shell fibers compared with the monolithic fibers, the interactions between PVA and the TSC are not very pronounced.

**Figure 4:**
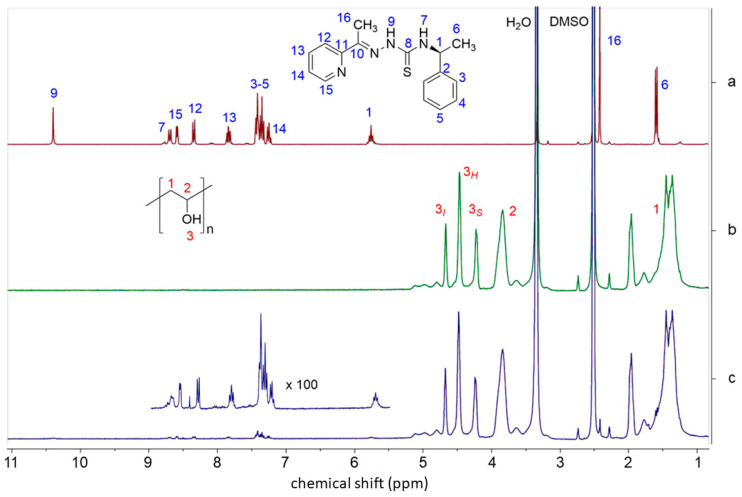
300 MHz ^1^H NMR spectra of (**a**) HapyTSCmB, (**b**) PVA, and (**c**) PVA/TSC10-PVA fibers in dimethyl sulfoxide (DMSO-*d*_6_) solution.

For electrospinning, a high voltage source generates an electric field between a droplet of the polymer solution and the collector plate [36], and these high-energy conditions might damage materials [73,74]. Therefore, the chemical integrity of PVA and HapyTSCmB after the electrospinning process was evaluated using NMR spectroscopy of dissolved fibers. The full ^1^H NMR spectrum of HapyTSCmB is shown in Figure 4a (data in the Materials and Methods section) alongside with spectra of PVA and the core–shell PVA/TSC10-PVA fibers dissolved in DMSO-d_6_.

The ^1^H NMR spectrum of dissolved PVA (Figure 4b) shows a signal triad at 4.66, 4.45, and 4.20 ppm assignable to the OH groups for iso-, hetero-, and atactic polymer branches, respectively [75,76,77,78]. The multiplets observed at 3.83 ppm and 1.95 to 1.40 ppm are attributed to the CH and CH_2_, respectively [75,76,77,78]. The large signals at 3.3 and 2.5 are due to H_2_O and DMSO-d_5_. In the ^1^H NMR spectrum of the PVA/TSC10-PVA fibers (Figure 4c), the characteristic resonances of the PVA polymer and the HapyTSCmB were found to be unchanged, confirming their chemical integrity and stability during the electrospinning process.

**Figure 5:**
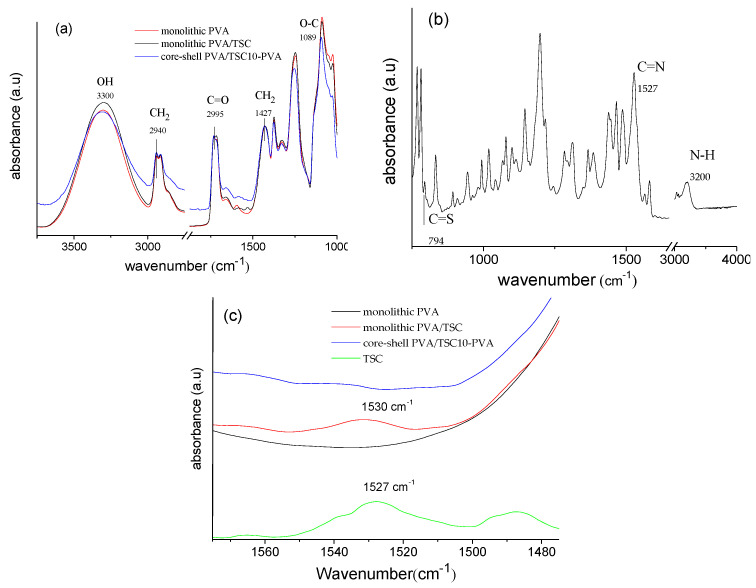
Normalized attenuated total reflection Fourier-transform infrared (ATR-FTIR) spectra of (**a**) monolithic PVA and PVA/TSC5 fiber mats. (**b**) HapyTSCmB, (**c**) monolithic PVA, PVA/TSC5, and core–shell PVA/TSC10-PVA fibers. The spectra were normalized based on the 1427 cm^−1^ CH_2_ bending vibration.

The ATR-FTIR spectra of all three fibers (Figure 5) showed large bands between 3600 and 3200 for all three samples. They can be assigned to the O–H stretching modes for the intermolecular and intramolecular hydrogen bonds in PVA (Figure 5a). Other bands characteristic for the electrospun PVA fibers were observed at 2940 cm^−1^ (asymmetric C–H stretching of the alkyl groups), 1731 cm^−1^ (C=O stretch), 1427 cm^−1^ (CH_2_ bending), and 1089 cm^−1^ (C–O–C bending) [76,77,78,79]. Importantly, there were no marked differences between monolithic pure PVA and PVA/TSC fibers in this spectral range. For pure HapyTSCmB (Figure 5b), we observed a band at 794 cm^−1^ related to the C=S stretching mode [80,81]. The intense band at 1527 cm^−1^ can be assigned to the vibration C=N group and the band at 3200 cm^−1^ can be assigned to N–H stretching vibrations [80,81,82]. The monolithic PVA/TSC5 fibers showed the TSC-specific band of the C=N vibration at 1531 cm^−1^, while for the PVA/TSC10-PVA core–shell fibers, this band was not observed (Figure 5c). We explain this with the limited penetration depths of the method, which might not allow observing the TSC in the core of the PVA/TSC10-PVA core–shell material but only TSC molecules close to the surface of the monolithic fibers. The relative low intensity points to a homogeneous distribution of the TSC in the monolithic fibers and no accumulation on the surface. For the PVA/TSC10-PVA core–shell fibers, the missing band is a strong support for the assumed core–shell structure.

### 3.2. Release Studies

**Figure 6:**
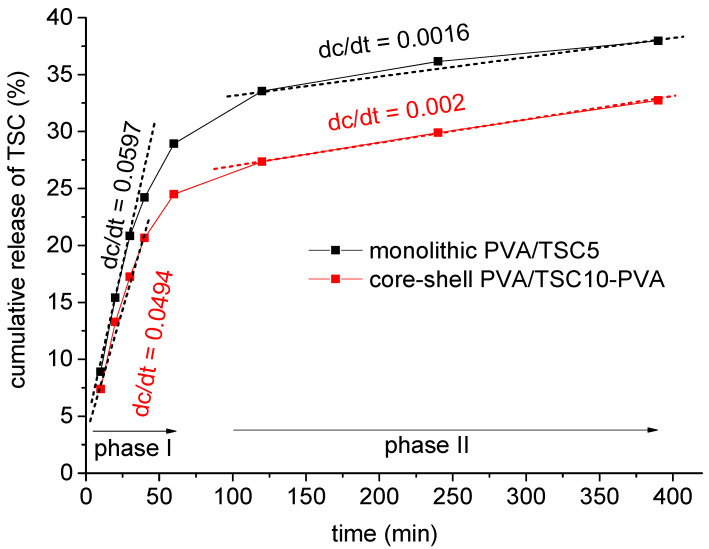
Cumulative release percentage of HapyTSCmB from the monolithic PVA/TSC5 and core–shell PVA/TSC10-PVA fiber mats over a period of 390 min.

The cumulative release of HapyTSCmB from the monolithic PVA/TSC5 and core–shell PVA/TSC10-PVA fiber mats into phosphate buffer solution (PBS, pH = 7.4) was studied during 390 min with a total release of about 38% and 33% release for PVA/TSC5 and PVA/TSC10-PVA, respectively (Figure 6) after 390 min. The release curves were fitted assuming an initial release phase from the beginning until about 60 min and a second release phase from 100 to 400 min. For the initial release of about 25–30%, which is rather a burst release [83,84], the rate is slightly higher for the monolithic PVA/TSC5 (0.6 versus 0.5). For the slower and sustained release phase, no plateau is reached after 390 h, and now, the release rate for the core–shell PVA/TSC10-PVA fibers is slightly higher (0.0020 versus 0.0016).

In the beginning, the PVA shell slightly retarded the release from the core–shell fibers compared with the monolithic fibers, but the first values were not much smaller for the core–shell fibers, and although the initial release rate was high for the monolithic PVA/TSC5 fibers, it was not much higher than found for the core–shell fibers. In view of the rather hydrophobic nature of the TSC and the hydrophilic character of PVA, we assume that the pure PVA shell of the core–shell fibers dissolved more rapidly in the PBS solution than the TSC-containing PVA/TSC5 fibers. Thus, the release of TSC from the core–shell fibers occurred already after a short period of time. This behavior also points to a homogeneous distribution of the TSC in the monolithic PVA/TSC5 fibers. If the TSC molecules were segregated on the surface of the PVA/TSC5 fibers, their rather hydrophobic character would probably hamper the solubility of the fibers in the PBS solution. In this case, we would expect strongly retarded release in the beginning and a burst release to almost completeness within a short period of time. A homogeneous distribution of the TSC in the monolithic PVA/TSC5 fibers is in agreement with the observed sustained release of only 38% within 390 min.

Compared to the reported release of drugs from polymers with similar hydrophilic/hydrophobic character [45,46,53,54,55,56,57], our release system combining a very hydrophobic drug (HapyTSCmB) with a very hydrophilic and water soluble polymer (PVA) is not very advanced, and the core–shell approach did not much improve the problem. Nevertheless, the release behavior of both materials might be suitable for potential medical applications, since the goal of shielding the TSC drug-containing core initially completely from the surrounding media with a PVA shell was achieved.

The release kinetics of HapyTSCmB from the PVA mats were studied fitting the experimental cumulative release data (*Q = m_t_/m_0_*) during the two release phases (phase I: from 0 to 60 min and phase II: from 100 to 400 min) to zero-order, first-order, Higuchi, and Korsmeyer–Peppas kinetic models. Then, the regression coefficients (*R^2^*) of different mathematical release models and the release exponent values *n* of the Korsmeyer–Peppas model were calculated from the model fitting on experimental release data (Table 2).

The best fit was obtained using the Korsmeyer–Peppas equation $Q=K_{K}t^{n}$, which is applicable to cylindrical samples [85] with *R^2^* values 0.99 and 0.98 in phase I and exponents *n* of 0.67 and 0.68 for PVA/TSC5 and PVA/TSC10-PVA, respectively (Table 2). For phase II, *R^2^* values of 0.97 and 0.99 were obtained and exponents of 0.14 and 0.15 were obtained as well, respectively. Values for phase I lying between 0.45 and 0.89 indicate that the release of TSC from the PVA mats represents anomalous transport diffusion [83,84,85,86,87], which is probably governed by a combination of TSC diffusion and the swelling of PVA fibers, which happened immediately upon contact with the buffer solution. In contrast to this, the *n* values in phase II are markedly lower than 0.45, which is consistent with a Fick-type diffusion mechanism [83,84,85,86,87].

### 3.3. Growth Inhibition Studies–Minimal Inhibitory Concentration

*Staphylococcus aureus* bacteria are found on the skin and mucous membranes, and humans are the major reservoir for these organisms. It is a significant human pathogen that causes several diseases, including skin infections, scalded-skin syndrome, toxic shock syndrome, endocarditis, septic arthritis, and osteomyelitis. The treatment of *Staphylococcus aureus* infections depends largely on the type of infection as well as the presence or absence of drug-resistant strains.

**Figure 7:**
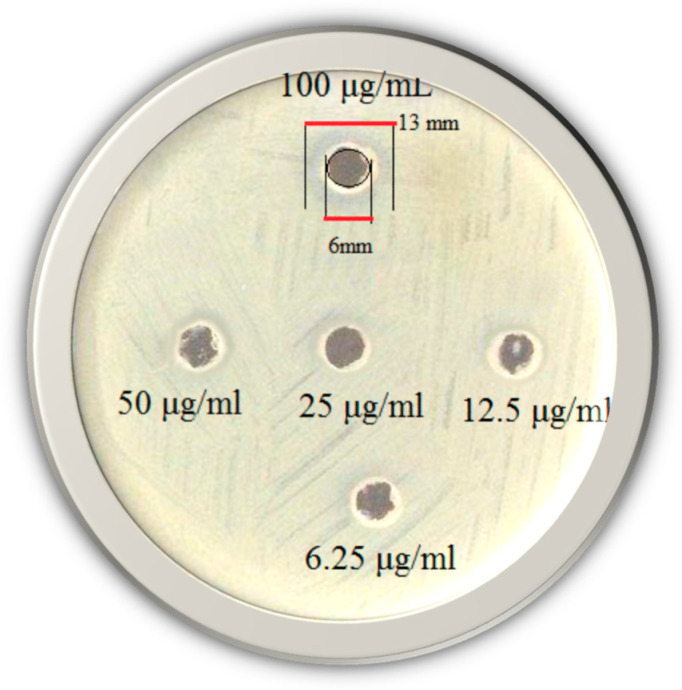
Photographic images of HapyTSCmB’s ability to inhibit the bacterial growth at concentrations of 6.25–100 μg/mL against *Staphylococcus aureus*.

Growth inhibition of the TSC against Gram-positive *Staphylococcus aureus* bacteria was screened in vitro at concentrations of 6.25–100 μg/mL using the agar well diffusion method (Figure 7) and a minimal inhibitory concentration (MIC) of 12.5 µg/mL was observed.

Thus, the HapyTSCmB showed good bacterial inhibitory property compared with other TSC compounds [59,80,81,82]. For TSCs and their metal complexes, MIC values from 10 up to 600 μg/mL against *Staphylococcus aureus* were reported [88,89,90]. A very low MIC of 1.5 μg/mL was reported for the copper complex of 1-phenyl-3-methyl-4-benzoyl-5-pyrazolone 4-ethyl-thiosemicarbazone [89]. Accordingly, the HapyTSCmB ligand represents medium antibacterial properties within the class of TSC compounds.

**Figure 8:**
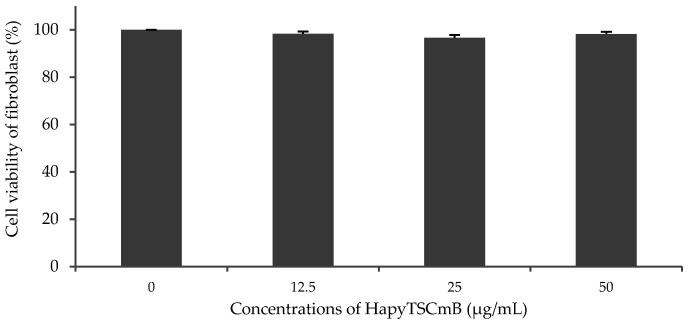
Cell viability of fibroplast cell incubated with increasing amounts of HapyTSCmB.

The cell viability of fibroblast cells was examined from 12.5 up to the 50 μg/mL of HapyTSCmB, based on the results of antibacterial properties and the MIC value (12.5 μg/mL). The cell viability of fibroblasts after 24 h in all samples containing HapyTSCmB was only slightly decreased by 1.5% up to 3.5% when compared to control cells (Figure 8). HapyTSCmB seems to have no cytotoxic properties on fibroblast cells and therefore seems to be suitable for use in drug-releasing electrospun fiber mats to treat herpes simplex infection.

## 4. Conclusions

PVA electrospun fiber mats containing the thiosemicarbazone (TSC) *N*4-(S)-1-phenylethyl-2-(pyridin-2-ylethylidene)hydrazine-1-carbothioamide (HapyTSCmB) were fabricated using a coaxial electrospinning setup device. Using different TSC loads to the spinning solutions (0, 5, and 10 wt%), we prepared core–shell PVA/TSC10-PVA) fibers containing 10% TSC in the core and for comparison monolithic fibers from core and shell solutions containing each 5 wt% TSC (PVA/TSC5) alongside pure monolithic PVA fibers. All three samples showed uniform and bead-free fibers with a cylindrical shape, smooth surfaces, and approximately the same size in scanning electron microscopy (SEM). The average diameter of the core–shell PVA/TSC10-PVA fibers is only slightly decreased (approximately 4%) compared with the pure PVA fibers. Initial experiments showed that addition of 10 wt% TSC to the spinning PVA solution caused a slight increase (approximately 6.5%) in conductivity and a significant decrease of about 83% in viscosity. A reduction in fiber size is generally in line with the decrease in viscosity; however, we are amazed that the size reduction is that small. DTA, TG, and DTG of the fibers in comparison to TG/DTA of the bulk TSC show similar behavior for both fibers such as evaporation of humidity, glass transition (*T*_g_), melting (*T*_m_), evaporation of the TSC, decomposition of the polymer side chains, and finally decomposition of the main polymer chain with increasing temperature for both monolithic TSC-free fibers as for PVA/TSC-PVA core–shell fibers. *T*_g_, *T*_m_, and the decomposition *T* of the core–shell PVA/TSC10-PVA fibers were slightly elevated compared with pure monolithic PVA fibers, which is in line with a re-enforcement caused by the TSC. The ^1^H NMR spectroscopy of dissolved PVA/TSC10-PVA fibers showed that the chemical integrity of HapyTSCmB and PVA was preserved during the electrospinning process. The C=N vibration observed at 1531 cm^−1^ in the ATR-FTIR spectra of the TSC was not found for the core–shell PVA/TSC10-PVA fibers, confirming that the PVA/TSC core was completely covered by the PVA shell.

After an initial fast release of HapyTSCmB of about 25–30% in 60 min, a slow and sustained release is found in the next 330 min for both types of fibers with a further cumulative release of about 10%. Both materials are basically suitable for drug release from their overall release behavior when looking at the release rates. However, the core–shell PVA/TSC-PVA fibers showed not only a generally slower release, but they also benefit from the effective shielding of the core-contained TSC (drug) through the PVS shell. This underlines that the core–shell approach can improve the problem of very different hydrophilic/hydrophobic character for the drug and the polymer and the release behavior of both materials might be suitable for potential medical applications. Nevertheless, slightly less hydrophilic polymers such as poly caprolactone (PCL) and slightly less hydrophobic TSC derivatives might yield systems allowing for controlled release instead of the retarded release reported here.

Following the Korsmeyer–Peppas release model, which was suited best to fit the experimental data, the release exponent values *n* for Phase I lying between 0.43 and 0.89 indicate anomalous transport diffusion, resulting from a combination PVA shell dissolution, TSC diffusion, and swelling of the fibers, while the *n* values in phase II lower than 0.43 are consistent with a Fick-type diffusion mechanism.

HapyTSCmB induced inhibition zones against the Gram-positive *Staphilococcus aureus* bacterium at the lowest content concentration (MIC) of 12.5 μg/mL and thus good bacterial inhibitory properties compared with other thiosemicarbazones compounds. MTT-based cytotoxicity results showed that the cell viability of fibroblast cells at concentrations of HapyTSCmB varying from 12.5 to 50 µg/mL had little to no influence on the cell viability when compared to control cells. This makes this TSC suitable for medical purposes such as the treatment of bacterial diseases.

## Figures and Tables

**Table 1:** Essential parameters for the fabrication of PVA and PVA/TSC fibers ^a^.

Sample Code	Method ^b^	HapyTSCmB Content (wt% Relative to PVA)			
		Fibers ^c^		Spinning Solutions	
		core	shell	core	shell
PVA	Method II/monolithic	0	0	PVA	PVA
PVA/TSC5	Method II/monolithic	5	5	PVA/TSC-5%	PVA/TSC-5%
PVA/TSC10-PVA	Method I/core-shell	10	0	PVA/TSC-10%	PVA

^a^ Feeding rates of all solutions: 0.3 mL/h. ^b^ See Scheme 2. ^c^ Assumed TSC content in the fibers.

**Table 2:** Calculated regression coefficients (*R*^2^) of different mathematical release models for the HapyTSCmB release kinetics from the PVA fiber mats ^a^.

Sample	$\mathrm{Zero}\;\mathrm{Order}\;\;Q=K_{0}t$		$\mathrm{First}\;\mathrm{Order}\;\;Q=1-e^{-K_{1}t}$		$\mathrm{Higuchi}\;\;Q=K_{H}t^{\frac{1}{2}}$		$\mathrm{Korsmeyer-Peppas}\;Q=K_{K}t^{n}$	
	Phase I *R*2	Phase II *R*^2^	Phase I *R*^2^	Phase II *R*^2^	Phase I *R*^2^	Phase II *R*^2^	Phase I *R*^2^	Phase II *R*^2^
PVA/TSC5	0.71	0.85	0.83	0.86	0.98	0.88	0.99	0.97
							*n* = 0.67	*n* = 0.14
PVA/TSC10-PVA	0.72	0.96	0.72	0.97	0.98	0.98	0.98	0.99
							*n* = 0.68	*n* = 0.15

^a^*Q = m_t_/m*_0_ is the experimentally obtained fraction of total released TSC up to time (*t_i_*), *K* is the kinetic constant of each model, and *n* is the release exponent.

## References

[1] Bahojb Noruzi E., Kheirkhahi M., Shaabani B., Geremia S., Hickey N., Asaro F., Nitti P., Kafil H.S. (2019). Design of a thiosemicarbazide-functionalized Calix[4]arene ligand and related transition metal complexes: Synthesis, characterization, and biological studies. Front. Chem..

[2] Bonaccorso C., Marzo T., La Mendola D. (2020). Biological applications of thiocarbohydrazones and their metal complexes: A perspective review. Pharmaceuticals.

[3] Scarim C.B., Jornada D.H., Machado M.G.M., Ferreira C.M.R., dos Santos J.L., Chung M.C. (2019). Thiazole, thio and semicarbazone derivatives against tropical infective diseases: Chagas disease, human African trypanosomiasis (HAT), leishmaniasis, and malaria. Eur. J. Med. Chem..

[4] Pelosi G. (2010). Thiosemicarbazone metal complexes: From structure to activity. Open Crystallogr. J..

[5] de Siqueira L.R.P., de Moraes Gomes P.A.T., de Lima Ferreira L.P., de Melo Rêgo M.J.B., Leite A.C.L. (2019). Multi-target compounds acting in cancer progression: Focus on thiosemicarbazone, thiazole and thiazolidinone analogues. Eur. J. Med. Chem..

[6] He Z., Qiao H., Yang F., Zhou W., Gong Y., Zhang X., Wang H., Zhao B., Ma L., Liu H.-M. (2019). Novel thiosemicarbazone derivatives containing indole fragment as potent and selective anticancer agent. Eur. J. Med. Chem..

[7] Akbari A., Ghateazadeh H., Takjoo R., Sadeghi-Nejad B., Mehrvar M., Mague J.T. (2019). Synthesis & crystal structures of four new biochemical active Ni(II) complexes of thiosemicarbazone and isothiosemicarbazone-based ligands: In vitro antimicrobial study. J. Mol. Struct..

[8] Volynets G.P., Tukalo M.A., Bdzhola V.G., Derkach N.M., Gumeniuk M.I., Tarnavskiy S.S., Starosyla S.A., Yarmoluk S.M. (2019). Benzaldehyde thiosemicarbazone derivatives against replicating and nonreplicating Mycobacterium tuberculosis. J. Antibiot..

[9] Pingaew R., Prachayasittikul S., Ruchirawat S. (2010). Synthesis, cytotoxic and antimalarial activities of benzoyl thiosemicarbazone analogs of isoquinoline and related compounds. Molecules.

[10] Pelosi G., Bisceglie F., Bignami F., Bignami P., Schiavone P., Re M.C., Casoli C., Pilotti E. (2010). Antiretroviral activity of thiosemicarbazone metal complexes. J. Med. Chem..

[11] Carradori S., Secci D., D’Ascenzio M., Chimenti P., Bolasco A. (2014). Microwave and ultrasound-assisted synthesis of thiosemicarbazones and their corresponding (4,5-substituted-thiazol-2-yl)hydrazines. J. Heterocycl. Chem..

[12] Klayman D.L., Scovill J.P., Bartosevich J.F., Bruce J. (1983). 2-acetylpyridine thiosemicarbazones. 5. 1-[1-(2-Pyridyl)ethyl]-3-thiosemicarbazides as potential antimalarial agents. J. Med. Chem..

[13] Katz E. (1987). Thiosemicarbazones: Inhibition of the growth of pox viruses and requirement for the growth of an isatin-β-thiosemicarbazone dependent mutant. J. Basic Clin. Physiol. Pharmacol..

[14] Kang I.J., Wang L.W., Hsu T.A., Yueh A., Lee C.C., Lee Y.C., Lee C.Y., Chao Y.S., Shih S.R., Chern J.H. (2011). Isatin-β-thiosemicarbazones as potent herpes simplex virus inhibitors. Bioorganic Med. Chem. Lett..

[15] Shipman C., Smith S.H., Drach J.C., Klayman D.L. (1981). Antiviral activity of 2-acetylpyridine thiosemicarbazones against herpes simplex virus. Antimicrob. Agents Chemother..

[16] Levinson W., Coleman V., Woodson B., Rabson A., Lanier J., Whitcher J., Dawson C. (1974). Inactivation of herpes simplex virus by thiosemicarbazones and certain cations. Antimicrob. Agents Chemother..

[17] Genova P., Varadinova T., Matesanz A.I., Marinova D., Souza P. (2004). Toxic effects of bis(thiosemicarbazone) compounds and its palladium(II) complexes on herpes simplex virus growth. Toxicol. Appl. Pharmacol..

[18] Shipman C., Smith S.H., Drach J.C., Klayman D.L. (1986). Thiosemicarbazones of 2-acetylpyridine, 2-acetylquinoline, 1-acetylisoquinoline, and related compounds as inhibitors of herpes simplex virus in vitro and in a cutaneous herpes guinea pig model. Antivir. Res..

[19] Turk S.R., Shipman C., Drach J.C. (1986). Structure-activity relationships among α-(N)-heterocyclic acyl thiosemicarbazones and related compounds as inhibitors of herpes simplex virus type 1-specified ribonucleoside diphosphate reductase. J. Gen. Virol..

[20] Altun A., Kumru M., Dimoglo A. (2001). The role of conformational and electronic parameters of thiosemicarbazone and thiosemicarbazide derivatives for their dermal toxicity. J. Mol. Struct. THEOCHEM.

[21] Altun A., Kumru M., Dimoglo A. (2001). Study of electronic and structural features of thiosemicarbazone and thiosemicarbazide derivatives demonstrating anti-HSV-1 activity. J. Mol. Struct. THEOCHEM.

[22] Savjani K.T., Gajjar A.K., Savjani J.K. (2012). Drug solubility: Importance and enhancement techniques. ISRN Pharm..

[23] Zhang X., Xing H., Zhao Y., Ma Z. (2018). Pharmaceutical dispersion techniques for dissolution and bioavailability enhancement of poorly water-soluble drugs. Pharmaceutics.

[24] Bajaj H., Bisht S., Yadav M., Singh V. (2011). Bioavailability enhancement: A review. Int. J. Pharma Bio Sci..

[25] Rao V.M., Sanghvi R., Zhu H. (2017). Solubility of pharmaceutical solids. Developing Solid Oral Dosage Forms.

[26] Serajuddin A.T.M. (1999). Solid dispersion of poorly water-soluble drugs: Early promises, subsequent problems, and recent breakthroughs. J. Pharm. Sci..

[27] Tran P., Pyo Y.C., Kim D.H., Lee S.E., Kim J.K., Park J.S. (2019). Overview of the manufacturing methods of solid dispersion technology for improving the solubility of poorly water-soluble drugs and application to anticancer drugs. Pharmaceutics.

[28] Xue J., Wu T., Dai Y., Xia Y. (2019). Electrospinning and electrospun nanofibers: Methods, materials, and applications. Chem. Rev..

[29] Islam M.S., Ang B.C., Andriyana A., Afifi A.M. (2019). A review on fabrication of nanofibers via electrospinning and their applications. SN Appl. Sci..

[30] Barani H. (2014). Antibacterial continuous nanofibrous hybrid yarn through in situ synthesis of silver nanoparticles: Preparation and characterization. Mater. Sci. Eng. C.

[31] Maleki H., Gharehaghaji A.A., Toliyat T., Dijkstra P.J. (2016). Drug release behavior of electrospun twisted yarns as implantable medical devices. Biofabrication.

[32] Singh A., Singh N. (2017). Recent review on nanofiber for drug delivery systems. World J. Pharm. Res..

[33] Pereira E.D., Cerruti R., Fernandes E., Peña L., Saez V., Pinto J.C., Ramón J.A., Oliveira G.E., de Souza Júnior F.G. (2016). Influence of PLGA and PLGA-PEG on the dissolution profile of oxaliplatin. Polímeros.

[34] Vasita R., Mani G., Agrawal C.M., Katti D.S. (2010). Surface hydrophilization of electrospun PLGA micro-/nano-fibers by blending with Pluronic^®^ F-108. Polymer (Guildford).

[35] Evrova O., Hosseini V., Milleret V., Palazzolo G., Zenobi-Wong M., Sulser T., Buschmann J., Eberli D. (2016). Hybrid randomly electrospun poly(lactic-co-glycolic acid):poly(ethylene oxide) (PLGA:PEO) fibrous scaffolds enhancing myoblast differentiation and alignment. ACS Appl. Mater. Interfaces.

[36] Teo W.-E., Inai R., Ramakrishna S. (2011). Technological advances in electrospinning of nanofibers. Sci. Technol. Adv. Mater..

[37] Pillay V., Dott C., Choonara Y.E., Tyagi C., Tomar L., Kumar P., du Toit L.C., Ndesendo V.M.K. (2013). A review of the effect of processing variables on the fabrication of electrospun nanofibers for drug delivery applications. J. Nanomater..

[38] Liechty W.B., Kryscio D.R., Slaughter B.V., Peppas N.A. (2010). Peppas, polymers for drug delivery systems. Annu. Rev. Chem. Biomol. Eng..

[39] Hu X., Liu S., Zhou G., Huang Y., Xie Z., Jing X. (2014). Electrospinning of polymeric nanofibers for drug delivery applications. J. Control. Release.

[40] Vlachou M., Siamidi A., Kyriakou S. (2019). Electrospinning and drug delivery. Electrospinning and Electrospraying—Techniques and Applications.

[41] Yu D.-G., Li J.-J., Williams G.R., Zhao M. (2018). Electrospun amorphous solid dispersions of poorly water-soluble drugs: A review. J. Control. Release.

[42] Imani R., Yousefzadeh M., Nour S. (2019). Functional nanofiber for drug delivery applications. Handbook of Nanofibers.

[43] Haider A., Haider S., Kang I.-K. (2018). A comprehensive review summarizing the effect of electrospinning parameters and potential applications of nanofibers in biomedical and biotechnology. Arab. J. Chem..

[44] Akhgari A., Shakib Z., Sanati S. (2017). A review on electrospun nanofibers for oral drug delivery. Nanomed. J..

[45] Buzgo M., Mickova A., Rampichova M., Doupnik M. (2018). Blend electrospinning, coaxial electrospinning, and emulsion electrospinning techniques. Core-Shell Nanostructures for Drug Delivery and Theranostics.

[46] Cornejo Bravo J.M., Villarreal Gómez L.J., Serrano-Medina A. (2016). Electrospinning for drug delivery systems: Drug incorporation techniques. Electrospinning—Material, Techniques, and Biomedical Applications.

[47] Teixeira M.A., Amorim M.T.P., Felgueiras H.P. (2019). Poly(vinyl alcohol)-based nanofibrous electrospun scaffolds for tissue engineering applications. Polymers (Basel).

[48] Bhattarai R.S., Bachu R.D., Boddu S.H.S., Bhaduri S. (2019). Biomedical applications of electrospun nanofibers: Drug and nanoparticle delivery. Pharmaceutics.

[49] Teodorescu M., Bercea M., Morariu S. (2018). Biomaterials of poly(vinyl alcohol) and natural polymers. Polym. Rev..

[50] Truong Y.B., Choi J., Mardel J., Gao Y., Maisch S., Musameh M., Kyratzis I.L. (2017). Functional cross-linked electrospun polyvinyl alcohol membranes and their potential applications. Macromol. Mater. Eng..

[51] Tang X., Alavi S. (2011). Recent advances in starch, polyvinyl alcohol based polymer blends, nanocomposites and their biodegradability. Carbohydr. Polym..

[52] Khalf A., Madihally S.V. (2017). Recent advances in multiaxial electrospinning for drug delivery. Eur. J. Pharm. Biopharm..

[53] Han D., Steckl A.J., Han D. (2019). Coaxial electrospinning formation of complex polymer fibers and their applications. ChemPlusChem.

[54] Qu H., Wei S., Guo Z. (2013). Coaxial electrospun nanostructures and their applications. J. Mater. Chem. A.

[55] Lu Y., Huang J., Yu G., Cardenas R., Wei S., Wujcik E.K., Guo Z. (2016). Coaxial electrospun fibers: Applications in drug delivery and tissue engineering. Wiley Interdiscip. Rev. Nanomed. Nanobiotechnology.

[56] Pant B., Park M., Park S.-J. (2019). Drug Delivery Applications of Core-Sheath Nanofibers Prepared by Coaxial Electrospinning: A Review. Pharmaceutics.

[57] Chou S.-F., Carson D., Woodrow K.A. (2015). Current strategies for sustaining drug release from electrospun nanofibers. J. Control. Release.

[58] Komiya S., Otsuka E., Hirashima Y., Suzuki A. (2011). Salt effects on formation of microcrystallites in poly(vinyl alcohol) gels prepared by cast-drying method. Prog. Nat. Sci. Mater. Int..

[59] Rodríguez-Argüelles M.C., Tourón-Touceda P., Cao R., García-Deibe A.M., Pelagatti P., Pelizzi C., Zani F. (2009). Complexes of 2-acetyl-γ-butyrolactone and 2-furancarbaldehyde thiosemicarbazones: Antibacterial and antifungal activity. J. Inorg. Biochem..

[60] Barani H., Montazer M., Samadi N., Toliyat T. (2011). Nano silver entrapped in phospholipids membrane: Synthesis, characteristics and antibacterial kinetics. Mol. Membr. Boil..

[61] Liu Y., Wang C. (2019). Advanced Nanofibrous Materials Manufacturing Technology Based on Electrospinning.

[62] Angammana C.J., Jayaram S.H. (2011). Analysis of the Effects of Solution Conductivity on Electrospinning Process and Fiber Morphology. IEEE Trans. Ind. Appl..

[63] Elkasaby M., Hegab H.A., Mohany A., Rizvi G.M. (2018). Modeling and optimization of electrospinning of polyvinyl alcohol (PVA). Adv. Polym. Tech..

[64] Rwei S.-P., Huang C.-C. (2012). Electrospinning PVA solution-rheology and morphology analyses. Fibers Polym..

[65] Okutan N., Terzi P., Altay F. (2014). Affecting parameters on electrospinning process and characterization of electrospun gelatin nanofibers. Food Hydrocoll..

[66] Yoon Y.I., Park K.E., Lee S.J., Park W.H. (2013). Fabrication of microfibrous and nano-/microfibrous scaffolds: Melt and hybrid electrospinning and surface modification of poly(L-lactic acid) with plasticizer. Biomed. Res. Int..

[67] Dasdemir M., Topalbekiroglu M., Demir A. (2013). Electrospinning of thermoplastic polyurethane microfibers and nanofibers from polymer solution and melt. J. Appl. Polym. Sci..

[68] Nazari T., Garmabi H. (2018). The effects of processing parameters on the morphology of PLA/PEG melt electrospun fibers. Polym. Int..

[69] Kim G.-M. (2010). Fabrication of bio-nanocomposite nanofibers mimicking the mineralized hard tissues via electrospinning process. Nanofibers.

[70] Wiśniewska M., Chibowski S., Urban T., Sternik D. (2011). Investigation of the alumina properties with adsorbed polyvinyl alcohol. J. Therm. Anal. Calorim..

[71] Fathi E., Atyabi N., Imani M., Alinejad Z. (2011). Physically crosslinked polyvinyl alcohol–dextran blend xerogels: Morphology and thermal behavior. Carbohydr. Polym..

[72] Jose T., George S.C., Maya M.G., Maria H.J., Wilson R., Thomas S. (2014). Effect of bentonite clay on the mechanical, thermal, and pervaporation performance of the poly(vinyl alcohol) nanocomposite membranes. Ind. Eng. Chem. Res..

[73] Thitiwongsawet P., Supaphol P. (2011). Carbendazim-loaded electrospun poly(vinyl alcohol) fiber mats and release characteristics of carbendazim therefrom. Polym. Adv. Technol..

[74] Wen P., Wen Y., Zong M.-H., Linhardt R.J., Wu H. (2017). Encapsulation of bioactive compound in electrospun fibers and its potential application. J. Agric. Food Chem..

[75] Korbag I., Saleh S.M. (2016). Studies on the formation of intermolecular interactions and structural characterization of polyvinyl alcohol/lignin film. Int. J. Environ. Stud..

[76] Negim E.-S., Bekbayeva L., Adam H., Yeligbayeva G., Ganjian E., Saleh M., Saad B. (2018). The effect of blend ratios on physico-mechanical properties and miscibility of cross-linked poly(vinyl alcohol)/urea blends. J. Phys. Conf. Ser..

[77] Sakurada I. (1985). Polyvinyl Alcohol Fibers.

[78] Moritani T., Kuruma I., Shibatani K., Fujiwara Y. (1972). Tacticity of poly(vinyl alcohol) studied by nuclear magnetic resonance of hydroxyl protons. Macromolecules.

[79] Mansur H.S., Sadahira C.M., Souza A.N., Mansur A.A.P. (2008). FTIR spectroscopy characterization of poly (vinyl alcohol) hydrogel with different hydrolysis degree and chemically crosslinked with glutaraldehyde. Mater. Sci. Eng. C.

[80] Chandra S., Usha (1992). Pd(II), Pt(II), Rh(III), Ir(III) and Ru(III) complexes of *n*-pentyl and *n*-hexyl ketone thiosemicarbazones. Synth. React. Inorg. Met. Chem..

[81] Ferraz K.S.O., Silva N.F., Da Silva J.G., Speziali N.L., Mendes I.C., Beraldo H. (2012). Structural studies on acetophenone- and benzophenone-derived thiosemicarbazones and their zinc(II) complexes. J. Mol. Struct..

[82] Offiong O.E. (1994). Synthesis and spectral studies of platinum metal complexes of benzoin thiosemicarbazone. Spectrochim. Acta Part A Mol. Spectrosc..

[83] Huang X., Brazel C.S. (2001). On the importance and mechanisms of burst release in matrix-controlled drug delivery systems. J. Control. Release.

[84] Mahmud M.M., Zaman S., Perveen A., Jahan R.A., Islam M.F., Arafat M.T. (2020). Controlled release of curcumin from electrospun fiber mats with antibacterial activity. J. Drug Deliv. Sci. Technol..

[85] Ritger P.L., Peppas N.A. (1987). A simple equation for description of solute release I. Fickian and non-Fickian release from non-swellable devices in the form of slabs, spheres, cylinders or discs. J. Control. Release.

[86] Natu M.V., de Sousa H.C., Gil M.H. (2010). Effects of drug solubility, state and loading on controlled release in bicomponent electrospun fibers. Int. J. Pharm..

[87] Sun Y., Cheng S., Lu W., Wang Y., Zhang P., Yao Q. (2019). Electrospun fibers and their application in drug controlled release, biological dressings, tissue repair, and enzyme immobilization. RSC Adv..

[88] Calatayud D.G., López-Torres E., Mendiola M.A. (2007). Diphenyllead(IV) chloride complexes with benzilthiosemicarbazones. The first bis(thiosemicarbazone) derivatives. Inorg. Chem..

[89] Khan S.A., Asiri A.M., Al-Amry K., Malik M.A. (2014). Synthesis, characterization, electrochemical studies, and in vitro antibacterial activity of novel thiosemicarbazone and its Cu(II), Ni(II), and Co(II) complexes. Sci. World J..

[90] Pahontu E., Julea F., Rosu T., Purcarea V., Chumakov Y., Petrenco P., Gulea A. (2015). Antibacterial, antifungal and in vitro antileukaemia activity of metal complexes with thiosemicarbazones. J. Cell. Mol. Med..

